# Noncommunicable diseases attributed to low levels of physical activity in Brazil: an epidemiologic Global Burden of Disease Study

**DOI:** 10.1590/1806-9282.20250203

**Published:** 2025-10-17

**Authors:** Erika da Silva Maciel, André Pontes-Silva, Francisco Winter dos Santos Figueiredo, Susana Carla Alves Franco, Fernando Rodrigues Peixoto Quaresma, Marcus Vinicius Nascimento-Ferreira

**Affiliations:** 1 Universidade Federal do Tocantins, Postgraduate Program in Teaching in Science and Health – Palmas (TO), Brazil.; 2 Universidade Federal de São Carlos, Department of Physical Therapy, Postgraduate Program in Physical Therapy – São Carlos (SP), Brazil.; 3 Escola Superior de Desporto de Rio Maior – Rio Maior, Portugal.; 4 Universidade Federal do Tocantins, Health, Physical Activity and Behavior Research (HEALTHY-BRA) Group – Miracema do Tocantins (TO), Brazil.

**Keywords:** Public health, Quality of life, Epidemiology

## Abstract

**OBJECTIVE:**

The aim of the study was to present estimates of mortality from noncommunicable diseases attributable to low physical activity in Brazil in 2019.

**METHODS:**

An epidemiologic and descriptive study. We retrieved the data during the month of September 2023. Two independent researchers accessed the indicators in the Global Burden of Disease database: (i) number of cases and (ii) mortality from cardiovascular diseases, diabetes, kidney diseases, and neoplasms, and (iii) level of physical activity in Brazilian individuals for the year 2019. Data were extracted by two researchers independently for the states of Brazil, stratified by sex (male and female), age groups (15–49 years, 50–69 years, and 70 or more years), cause of death and corresponding mortality (cardiovascular diseases, diabetes and kidney diseases, and neoplasms) and classified by regions according to geographic and administrative distribution in North, Northeast, Southeast, South, and Central-West. The number of deaths, age-standardized mortality, and years of life lost due to the disease were extracted, gross and in rates per 100,000 inhabitants.

**RESULTS:**

Mortality from noncommunicable diseases associated with low levels of physical activity in Brazil in 2019 was 293.39 deaths per 100,000 inhabitants, highest in Maranhão, with 407.98 deaths per 100,000 inhabitants, and lowest in the Distrito Federal and Minas Gerais, respectively.

**CONCLUSIONS:**

Cardiovascular disease was the most prominent risk factor among the results of this study.

## INTRODUCTION

The importance of physical activity for human health is widely supported by an extensive scientific base that highlights the benefits of reducing the risk of chronic disease^1–6^ and improving mental health, reducing symptoms of anxiety and depression, and promoting a sense of well-being^7–11^.

A public policy that increases the regular practice of physical activity is able to help reduce the cost of health treatments, since the promotion of physical activity in the Brazilian population to prevent early mortality has been indicated as a protective factor against early mortality, as in a study with data from the Global Burden of Disease (GBD) for Brazil and states between 1990 and 2017^1^.

Physical inactivity is an important modifiable risk factor for the occurrence of noncommunicable diseases and mental health problems, including stroke, hypertension, type 2 diabetes, coronary heart disease, several types of cancer, dementia, depression, and all-cause mortality^1^. It is estimated that 499.2 million new cases of chronic disease could occur worldwide by 2030 if physical activity patterns do not change, resulting in direct health care costs^1^.

To reduce these numbers, the World Health Organization (WHO) launched the Global Action Plan for Physical Activity in 2018, which aims to reduce physical inactivity among adults and adolescents by 15% by 2030. However, global trends in physical activity levels show that the world is not on track to meet the 2030 target^12^.

In addition, the Global Action Plan sets targets that were designed before the Covid-19 pandemic and may not be achievable today, given the need to adopt restrictive measures such as quarantine and social distancing, which inevitably promoted sedentary lifestyles in all age groups^13^.

In 2017, data from the GBD study estimated that physical inactivity was a risk factor for approximately 1.3 million deaths (17 deaths per 100,000 population) in individuals aged 25 years and older^14^. In 2018, data from the GBD indicated that physical inactivity was responsible for 1.6 million deaths worldwide^15,16^. However, no study has summarized mortality estimates from noncommunicable diseases attributable to low physical activity in Brazil in 2019.

Therefore, the aim of this study was to present estimates of mortality from noncommunicable diseases attributable to low physical activity in Brazil in 2019.

## METHODS

### Study design

An epidemiologic and descriptive study based on secondary data from the GBD study, developed and maintained by the Institute of Health Metrics and Evaluation, University of Washington, United States. The standardized analytical methodology used to compare GBD data across countries, regions, and subnational data, including trend analysis, has been published elsewhere^17^.

### Ethical aspects

The data used in this study are publicly available, and it is not possible to access information that identifies the participants. In these cases, according to Resolution Number 510 of 2016 of the National Health Council, there is no indication of submission or review by a research ethics committee^18^.

### Database

We retrieved the data during the month of September 2023. Two independent researchers accessed the indicators in the GBD database (https://vizhub.healthdata.org/gbd-results/): (i) number of cases, (ii) mortality from cardiovascular diseases, diabetes, kidney diseases, and neoplasms, and (iii) level of physical activity in Brazilian individuals for the year 2019.

The GBD platform receives international collaboration from researchers from different parts of the world and makes data available at different regional levels, ranging from global statistics to data for Brazilian federal units, for example. Brazil is a country of continental proportions. And in 2019, the Brazilian Institute of Geography and Statistics (IBGE) estimated a population of 210.01 million inhabitants.

The country is divided into five macro-regions: North, Northeast, South, Southeast, and Central-West. Each macro-region is made up of a number of Brazilian states, as it is also known. Each state of Brazil is a political and administrative division of the country. There are 26 states of Brazil that have autonomy to govern themselves in certain geographical areas; they are made up of 25 states and the Distrito Federal.

### Data extraction

Data were extracted by two researchers independently for the states of Brazil, stratified by sex (male and female), age groups (15–49 years, 50–69 years, and 70 or more years), cause of death and corresponding mortality (cardiovascular diseases, diabetes and kidney diseases, and neoplasms), and classified by regions according to geographic and administrative distribution in North, Northeast, Southeast, South, and Central-West. The number of deaths, age-standardized mortality, and years of life lost due to the disease were extracted, gross and in rates per 100,000 inhabitants.

### Outcomes

The level of physical activity reported in the GBD is a result of classification performed using the International Physical Activity Questionnaire^19^ and the Global Physical Questionnaire^20^, which are self-report instruments and estimate the amount of metabolic equivalents per minute that a person spends on their activities during a week, estimating the amount of metabolic equivalent task per minute per week, with a low level of physical activity classified by the presence of less than 3,000 metabolic equivalent tasks/min/week^21^.

Data for geographic units, sex, and other characteristics available for extraction are estimated using databases provided by researchers around the world through statistics that include DisMod models, among other validated and internationally accepted statistics^21^.

### Noncommunicable diseases and conditions attributed to low levels of physical activity

Estimates of noncommunicable diseases and conditions attributable to low physical activity were extracted from the platform and analyzed in this study. They are calculated using a formula that takes into account mortality from causes and the proportion of the population with low levels of physical activity found in data sources from published and unpublished studies and meta-analyses. The estimate is calculated for each age group, geographic location, and year and is available on the platform from which the data were extracted^21,22^.

### Statistical analysis

To access noncommunicable diseases attributed to low physical activity, we report the total number of deaths according to their respective distributions by outcome related to low physical activity. Age-adjusted mortality was extracted for each of the categories, including age groups, taking into account the age distributions in each of the analysis units studied. We used SPSS™ for analysis.

## RESULTS

In 2019, the most recent data available from the GBD, 45,559 cases of chronic noncommunicable diseases were attributed to low physical activity in Brazil, with 293.39 deaths per 100,000 people.

Cardiovascular diseases are the chronic noncommunicable diseases with the highest mortality potential, as well as living in the Northeast region, being male, and being 70 years of age or older (Table 1).

**Table 1:** Distribution of deaths and mortality profile from noncommunicable diseases attributed to low levels of physical activity (Brazil, 2019).

Variable		Noncommunicable diseases associated with low levels of physical activity		
		n	%	Mortality
Total		45,559	100	293.39
Sex				
	Female	24,894	54.6%	291.18
	Male	20,665	45.4%	295.25
Age (years)				
	15–49	2,215	4.9%	1.92
	50–69	11,649	25.6%	28.87
	70+	31,695	69.6%	242.16
Cause				
	Cardiovascular diseases	30,417	66.8%	188.42
	Diabetes and kidney disease	11,382	25.0%	63.41
	Neoplasms	4,032	8.9%	21.11
Region				
	North	2,620	5.7%	269.4
	Northeast	14,126	31.0%	329.9
	Southeast	19,398	42.6%	259.7
	Midwest	2,518	5.5%	229.8
	South	6,898	15.1%	264.6

**Figure 1:**
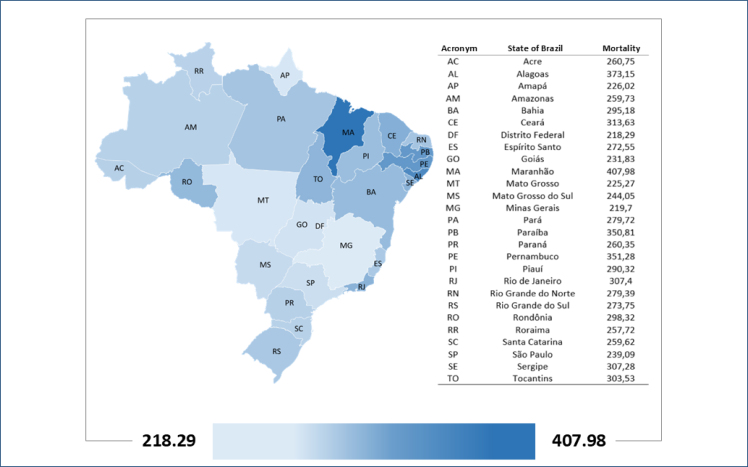
Geographic distribution of mortality from noncommunicable diseases attributed to low levels of physical activity (Brazil, 2019).

**Figure 2:**
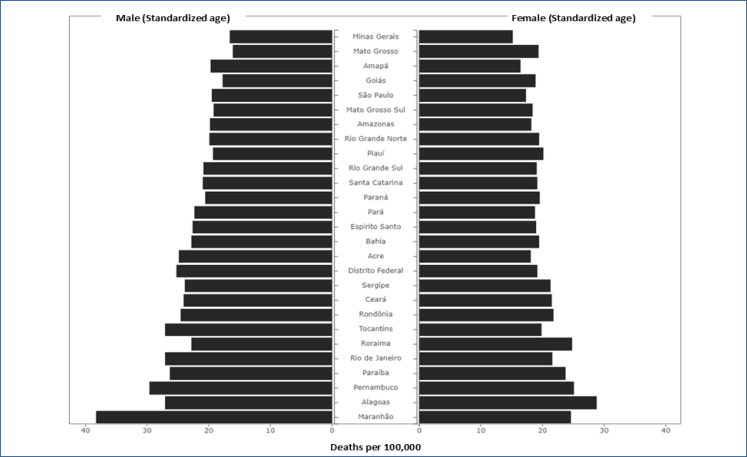
Mortality from chronic noncommunicable diseases attributed to low levels of physical activity in Brazilian states (Brazil, 2019).

In relation to the States of Brazil, it is observed that the mortality attributed to low levels of physical activity is highest in Maranhão, with 407.98 deaths per 100,000 inhabitants; the lowest mortality was found in the Distrito Federal, followed by Minas Gerais (Figures 1 and 2). Mortality in the Brazilian states varies from 218.9 to 407.98 deaths per 100,000 inhabitants.

Regarding the global burden of noncommunicable diseases attributed to low levels of physical activity, it is observed that the highest rates are found in men over 70 years of age with cardiovascular diseases in the Southeast region (Table 2).

**Table 2:** Global burden of noncommunicable diseases and conditions attributed to low levels of physical activity (Brazil, 2019).

Variable		Noncommunicable diseases associated with low levels of physical activity	
		Disability-adjusted life years	Rate*
Total		1,051,620.5	449
Sex			
	Female	541,221.9	417
	Male	510,398.5	486
Age (years)			
	15–49	150,801.1	131
	50–69	446,449.9	1,107
	70+	454,369.5	3,472
Cause			
	Cardiovascular diseases	538,080.4	233
	Diabetes and kidney disease	472,721.1	178
	Neoplasms	88,819.0	37
Region			
	North	65,222	334
	Northeast	314,176	506
	Southeast	453,529	523
	Midwest	65,118	394
	South	153,575	508

* Per 100,000 population, standardized by age except for estimates of rates by age group.

## DISCUSSION

Mortality from noncommunicable diseases and conditions attributable to low physical activity in Brazil in 2019 was 293.39 deaths per 100,000 inhabitants, and cardiovascular disease was the most prominent risk factor among the findings of this study.

In terms of Disability-Adjusted Life Years, a measure of population health that combines the burden of mortality and morbidity in a single metric, we found that men aged 50 and older with cardiovascular disease and residents of the Southeast region have a higher indicator of years lost due to premature death and disability related to insufficient physical activity.

In 2021, the WHO estimates that around 3.2 million deaths worldwide will be attributable to low levels of physical activity—including 9% of premature deaths^23^. About a decade ago, an analysis of studies conducted in Brazil highlighted the significant impact of low levels of physical activity on Brazilian mortality^24^.

In addition, the relationship between physical activity and cardiovascular health benefits has been studied since the 1950s, and the benefits of physical activity have been consistently demonstrated^25–27^. However, despite these globally recognized benefits, regular physical activity is not a reality for the majority of the world's population and tends to worsen in less developed countries^28^.

Mortality due to noncommunicable diseases is projected to rise from 38 million in 2012 to 52 million in 2030, and this group of diseases is increasing in low- and middle-income countries in tandem with the increase in physical inactivity in recent years^29^. Coronary heart disease, the major health problem of western life. A number of factors contribute to coronary heart disease, including high serum cholesterol levels, hypertension, smoking, and a sedentary lifestyle^30^.

In a longitudinal study of men and women who engaged in regular physical activity at least twice a week, they had a 41% lower risk of developing coronary heart disease than those who did not engage in physical activity, and the long-term beneficial effect of leisure-time physical activity on the risk of coronary heart disease in women and men remained even after controlling for income and other important risk factors for heart disease^31^.

In the results found in the present study, it is observed that states in the North and Northeast have a greater potential for exposure to physical inactivity. All-cause mortality due to physical inactivity in Brazil and Brazilian states over 28 years (1990–2017) was estimated in a previous study, and it was found that the Brazilian population was at risk of exposure to physical inactivity, contributing to a significant number of deaths in the period analyzed. Brazilian states with better socioeconomic conditions showed greater reductions in age-standardized mortality over the 28-year period^32^.

Although the historical study showed a reduction/stability in deaths from major chronic diseases associated with physical inactivity^32^. Mortality from diabetes and hypertension remains high, accounting for 83% of deaths in 2019^33^. Potentially the worst diet or a suboptimal diet and hypertension are associated with mortality from cardiovascular disease in 2019. In addition, economic inequality can help explain the worse nutritional indicators in the state of Maranhão, as well as the worse prevention, treatment, or even health diagnosis mechanisms^34^.

For example, in Maranhão (along with Pernambuco and Alagoas), the main cause of death is related to hypertension (and deaths from diabetes have a higher rate compared to other states in Brazil). Therefore, if among the main diseases, diabetes and hypertension are still high (they have not decreased as reported by others) and are the main causes in Maranhão, it is possible to assume that the economic disparity will reinforce the historical findings^17^, which accentuate our results. Maranhão has the worst (highest) deaths due to cardiovascular outcomes, while exposure to low levels of physical activity appears to be maintained^35^.

The results of the present study reinforce the importance of monitoring risk exposure and the usefulness of the GBD study in synthesizing data to draw comprehensive and robust conclusions that help guide effective policy and strategic planning in health promotion efforts^14^.

Implement community-based public education and physical activity awareness campaigns, including media campaigns, combined with other community-based, motivational, and environmental education programs, to support behavioral changes in physical activity levels^23^.

Low-cost interventions, such as walking, to improve the health of citizens are viable alternatives to increase physical activity levels in the population. In addition, schools, as one of the settings with which most children and adolescents interact, are favorable environments for promoting healthier and less costly lifestyles^29^. Likewise, the implementation of physical activity programs in health centers, as an object of primary health care in Brazil, is low-cost and has important coverage of society.

## CONCLUSION

Mortality from noncommunicable diseases associated with low levels of physical activity in Brazil in 2019 was 293.39 deaths per 100,000 inhabitants, highest in Maranhão, with 407.98 deaths per 100,000 inhabitants, and lowest in the Distrito Federal and Minas Gerais, respectively. Finally, cardiovascular disease was the most prominent risk factor among the results of this study.

## Data Availability

The datasets generated and/or analyzed during the current study are available from the corresponding author upon reasonable request.

## References

[1] Santos AC, Willumsen J, Meheus F, Ilbawi A, Bull FC (2023). The cost of inaction on physical inactivity to public health-care systems: a population-attributable fraction analysis. Lancet Glob Health.

[2] Silva DAS, Tremblay MS, Marinho F, Ribeiro ALP, Cousin E, Nascimento BR (2020). Physical inactivity as a risk factor for all-cause mortality in Brazil (1990-2017). Popul Health Metr.

[3] Maciel Eda S, Vilarta R, Modeneze DM, Sonati JG, Vasconcelos JS, Vilela GB (2013). The relationship between physical aspects of quality of life and extreme levels of regular physical activity in adults. Cad Saude Publica.

[4] Pitanga FJG, Matos SMA, Almeida MDC, Barreto SM, Aquino EML (2018). Leisure-time physical activity, but not commuting physical activity, is associated with cardiovascular risk among ELSA-Brasil participants. Arq Bras Cardiol.

[5] Hallal PC, Victora CG, Wells JC, Lima RC (2003). Physical inactivity: prevalence and associated variables in Brazilian adults. Med Sci Sports Exerc.

[6] Ding D, Lawson KD, Kolbe-Alexander TL, Finkelstein EA, Katzmarzyk PT, Mechelen W (2016). The economic burden of physical inactivity: a global analysis of major non-communicable diseases. Lancet.

[7] Gabriela A, Vilela S, Crisp AH (2020). Níveis de estresse, ansiedade, depressão e fatores associados durante a pandemia de COVID-19 em praticantes de Yoga.

[8] Campos CG, Muniz LA, Belo VS, Romano MCC, Lima MC (2019). Adolescents’ knowledge about the benefits of physical exercises to mental health. Cien Saude Colet.

[9] Fox KR (1999). The influence of physical activity on mental well-being. Public Health Nutr.

[10] Andrade AC, Peixoto SV, Friche AA, Goston JL, César CC, Xavier CC (2015). Social context of neighborhood and socioeconomic status on leisure-time physical activity in a Brazilian urban center: the BH health study. Cad Saude Publica.

[11] Mura G, Cossu G, Migliaccio GM, Atzori C, Nardi AE, Machado S (2014). Quality of life, cortisol blood levels and exercise in older adults: results of a randomized controlled trial. Clin Pract Epidemiol Ment Health.

[12] Sharma C, Ahuja KDK, Kulkarni B, Byrne NM, Hills AP (2023). Life course research in physical activity: pathway to global action plan 2030. Obes Rev.

[13] Amini H, Habibi S, Islamoglu AH, Isanejad E, Uz C, Daniyari H (2021). COVID-19 pandemic-induced physical inactivity: the necessity of updating the global action plan on physical activity 2018-2030. Environ Health Prev Med.

[14] GBD 2017 Risk Factor Collaborators (2018). Global, regional, and national comparative risk assessment of 84 behavioural, environmental and occupational, and metabolic risks or clusters of risks for 195 countries and territories, 1990-2017: a systematic analysis for the Global Burden of Disease Study 2017. Lancet.

[15] Ostro B, Spadaro JV, Gumy S, Mudu P, Awe Y, Forastiere F (2018). Assessing the recent estimates of the Global Burden of Disease for ambient air pollution: methodological changes and implications for low- and middle-income countries. Environ Res.

[16] GBD 2019 Diseases and Injuries Collaborators (2020). Global burden of 369 diseases and injuries in 204 countries and territories, 1990-2019: a systematic analysis for the Global Burden of Disease Study 2019. Lancet.

[17] Silva DAS, Tremblay MS, Marinho F, Ribeiro ALP, Cousin E, Nascimento BR (2020). Physical inactivity as a risk factor for all-cause mortality in Brazil (1990-2017). Popul Health Metr.

[18] Guerriero ICZ, Minayo MC (2019). The approval of resolution cns no. 510/2016 is a progress for Brazilian science. Saude Soc.

[19] Pardini R, Matsudo S, Matsudo V, Andrade E, Braggion G, Andrade D (2001). Validação do questionário internacional de nível de atividade física (IPAQ - versão 6): estudo piloto em adultos jovens brasileiros. Rev Bras Ciênc Mov.

[20] Armstrong T, Bull F (2006). Development of the World Health Organization Global Physical Activity Questionnaire (GPAQ). J Public Health (Bangkok).

[21] Yin X, Zhang T, Zhang Y, Man J, Yang X, Lu M (2022). The global, regional, and national disease burden of breast cancer attributable to low physical activity from 1990 to 2019: an analysis of the Global Burden of Disease Study 2019. Int J Behav Nutr Phys Act.

[22] Xu YY, Xie J, Yin H, Yang FF, Ma CM, Yang BY (2022). The Global Burden of Disease attributable to low physical activity and its trends from 1990 to 2019: an analysis of the Global Burden of Disease study. Front Public Health.

[23] Oliveira AB, Woldeamanuel YW, Kubota GT, Delgado PB, Pelicer YC, Partamian K (2025). Socioeconomic and lifestyle factors associated with chronic musculoskeletal disorders in Brazil: a network analysis of a population-based study involving 87,648 Brazilian adults. Ther Adv Chronic Dis.

[24] Hallal PC (2014). Physical activity and health in Brazil: research, surveillance and policies. Cad Saude Publica.

[25] Sesso HD, Paffenbarger RS, Ha T, Lee IM (1999). Physical activity and cardiovascular disease risk in middle-aged and older women. Am J Epidemiol.

[26] Ding D, Lee IM, Bauman AE, Ekelund U, Stamatakis E (2021). Dietary risk versus physical inactivity: a forced comparison with policy implications?. Lancet.

[27] Barengo NC, Hu G, Lakka TA, Pekkarinen H, Nissinen A, Tuomilehto J (2004). Low physical activity as a predictor for total and cardiovascular disease mortality in middle-aged men and women in Finland. Eur Heart J.

[28] Maciel EDS, Silva BKR, Figueiredo FWDS, Pontes-Silva A, Quaresma FRP, Adami F (2022). Physical inactivity level and lipid profile in traditional communities in the Legal Amazon: a cross-sectional study: physical inactivity level in the Legal Amazon. BMC Public Health.

[29] Liu W, Dostdar-Rozbahani A, Tadayon-Zadeh F, Akbarpour-Beni M, Pourkiani M, Sadat-Razavi F (2022). Insufficient level of physical activity and its effect on health costs in low- and middle-income countries. Front Public Health.

[30] Mozaffarian D, Benjamin EJ, Go AS, Arnett DK, Blaha MJ, Writing Group Members (2016). Heart disease and stroke statistics-2016 update: a report from the American Heart Association. Circulation.

[31] Sundquist K, Qvist J, Johansson SE, Sundquist J (2005). The long-term effect of physical activity on incidence of coronary heart disease: a 12-year follow-up study. Prev Med.

[32] Silva DAS, Tremblay MS, Marinho F, Ribeiro ALP, Cousin E, Nascimento BR (2020). Physical inactivity as a risk factor for all-cause mortality in Brazil (1990-2017). Popul Health Metr.

[33] Malta DC, Passos VMA, Vasconcelos AMN, Carneiro M, Gomes CS, Ribeiro ALP (2022). Disease burden in Brazil and its states. Estimates from the Global Burden of Disease Study 2019. Rev Soc Bras Med Trop.

[34] Brant LCC, Nascimento BR, Veloso GA, Gomes CS, Polanczyk C, Oliveira GMM (2022). Burden of cardiovascular diseases attributable to risk factors in Brazil: data from the "Global Burden of Disease 2019" study. Rev Soc Bras Med Trop.

[35] Silva DAS, Ribeiro ALP, Marinho F, Naghavi M, Malta DC (2022). Physical activity to prevent stroke mortality in Brazil (1990-2019). Rev Soc Bras Med Trop.

